# Evaluation of Various Factors Affecting Bioconversion of l-Tyrosine to l-DOPA by Yeast *Yarrowia lipolytica*-NCIM 3450 Using Response Surface Methodology

**DOI:** 10.1007/s13659-014-0017-3

**Published:** 2014-05-07

**Authors:** Swati T. Gurme, Shripad N. Surwase, Sushama A. Patil, Jyoti P. Jadhav

**Affiliations:** 1 Department of Biotechnology, Shivaji University, Vidyanagar, Kolhapur, 416004 India; 2 Department of Microbiology, Shivaji University, Kolhapur, 416004 India

**Keywords:** l-DOPA, l-tyrosine, RSM, *Yarrowia lipolytica*

## Abstract

**Abstract:**

3,4-Dihydroxy l-phenylalanine (l-DOPA) is considered a potent drug for the treatment of Parkinson disease. Physical and nutritional parameters where optimized by using *Yarrowia lipolytica*-NCIM 3450 to accomplished the highest production of l-DOPA. Screenings of critical components were completed by using a Plackett–Burman design, while further optimization was carried out using the Box–Behnken design. The optimized factor levels predicted by the model were pH 6.1, 1.659 g L^−1^ yeast extract, 1.491 g L^−1^l-tyrosine and 0.0290 g L^−1^ CuSO_4_. The predicted yield of l-DOPA with these levels was 1.319 g L^−1^, while actual yield obtained was 1.273 g L^−1^. The statistical analysis revealed that model is significant with F value 19.55 and R^2^ value 0.9514. This process resulted in a 3.594-fold increase in the yield of l-DOPA. l-DOPA was confirmed by HPTLC and HPLC analysis. Thus, *Yarrowia lipolytica*-NCIM 3450 has potential to be a new source for the production of l-DOPA.

**Graphical Abstract:**

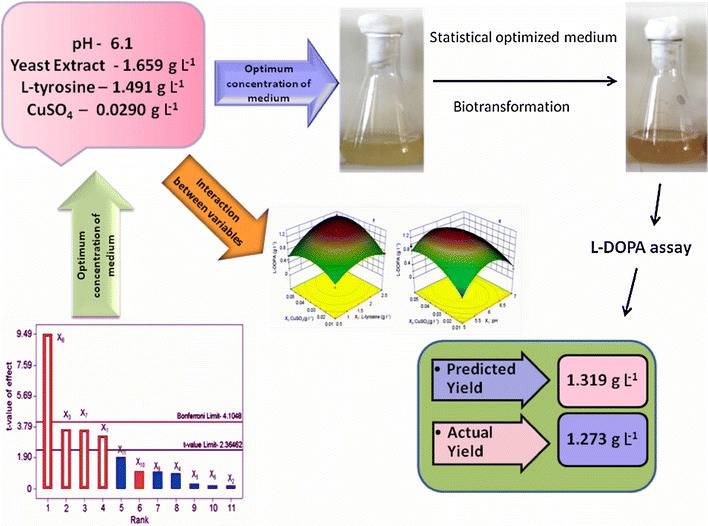

**Electronic supplementary material:**

The online version of this article (doi:10.1007/s13659-014-0017-3) contains supplementary material, which is available to authorized users.

## Introduction

Parkinson’s disease affects individuals worldwide, with the incidence increasing sharply with age to about 200–250 per 20 million in those over 60 years old. l-DOPA (3,4-dihydroxy phenyl l-alanine) is the drug of choice in the treatment of Parkinson’s disease and for controlling the changes in enzymes of energy metabolism in Myocardium following neurogenic injury [1]. l-DOPA is produced from l-tyrosine by one-step oxidation reaction by which is catalyzed by enzyme tyrosinase [2, 3]. Tyrosinases (EC 1.14.1.18.1) are widely distributed in Nature and have been purified to homogeneity from both microbial and plant sources [4].

About 250 tons of l-DOPA is now supplied per year with trade names Dopar, Larodopar, Sinemet, [5, 6]. As the demand for l-DOPA is high, its production by various biological sources is highly relevant [7]. l-DOPA have been produced earlier by several biological sources that include *Erwinia herbicola* [8], *Aspergillus oryzae* [9], *Yarrowia lipolytica* NRRL-143 [10]*, Bacillus* sp. JPJ [11] and *Brevundimonas* sp. SGJ [12]*, Acremonium rutilum* [13] and Egyptian halophilic black yeast [14]. In addition, plant sources, such as cell suspension cultures of banana and *Portulaca grandiflora*, have also been reported for l-DOPA production [15, 16]. The seeds of *M. pruriens* [17], *M. monosperma* [18] have been used for l-DOPA production. Most of the l-DOPA sold commercially is chemically synthesized that involves eight reaction steps. Chemical synthesis of l-DOPA is a time-consuming process which involves several chemicals that are extremely costly and requires catalysts that are not ecofriendly [13, 19]. In contrast to chemical production, biotechnological production of l-DOPA by microorganisms is environmental friendly and enables an enhanced product under simple process conditions [8].

The optimization of fermentation conditions, particularly physical and nutritional parameters are of primary importance in the development of any fermentation process owing to their impact on the economy and practicability of the process [20]. Classical method have some disadvantages like more time consumption, laborious process and high cost, in addition to this, it fails to determine the combined effect of different factors. Thus researchers are encouraged to apply statistical approaches such as ‘response surface methodology’ (RSM), which provide a great amount of information based on only a small number of experiments [21, 22]. In the present study Plackett–Burman design and Box–Behnken design of the RSM were used to optimize the medium compositions and cultivation conditions for the highest l-DOPA production by using *Y.* *lipolytica*-NCIM 3450.

## Results and Discussion

### Plackett–Burman Design for Screening of Critical Factors

**Fig. 1:**
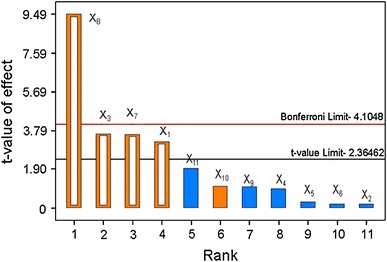
Pareto chart showing significant effects of factors above the ‘Bonferroni Limit’ and ‘t-value Limit’ and insignificant effect of the factors below the ‘Bonferroni Limit’ and ‘t-value Limit’ X_1_ (pH), X_2_ (temperature), X_3_ (yeast extract), X_4_ (peptone), X_5_ (beef extract), X_6_ (sucrose), X_7_ (l-tyrosine), X_8_ (CuSO_4_), X_9_ (MgSO_4_), X_10_ (K_2_HPO_4_), and X_11_ (Thiamine)

Statistical analysis using a Plackett–Burman design implies that pH (X_1_), yeast extract (X_3_), l-tyrosine (X_7_), and CuSO_4_ (X_8_) were significantly affected the l-DOPA production. The remaining components were found to be insignificant. The ‘Pareto chart’ (Fig. 1) showed that value of l-tyrosine (X_7_) was above the ‘Bonferroni Limit’, this indicates it is certainly significant. Also the values of pH (X_1_), yeast extract (X_3_), l-tyrosine (X_7_), and CuSO_4_ (X_8_) were above the *t* value limit that implies that these factors are possibly significant. While the remaining factors were below the *t*-value limit which indicates their insignificance [23]. Statistical analysis of the responses was performed, as shown in Table 1. The model F value of 31.7145 implies that the model is significant. The values of “prob > F” less than 0.05 indicate model terms are significant. “Adeq Precision” measures the signal-to-noise ratio, with a ratio greater than 4 regarded as desirable [23]. The “Adeq Precision” ratio of 9.007 obtained in this study indicates an adequate signal. Thus, this model can be used to navigate the design space. Statistical analysis showed that it is not possible to evaluate the relationship between significant independent variables and the response by a first-order equation. Thus, the first-order model is not appropriate to predict the response; hence the further investigation could be conducted through a second-order model.

**Table 1:** Statistical analysis of the model by Plackett–Burman design for l-DOPA production

Source	Sum of Squares	df	Mean square	F value	*P* value Prob > F
Model	0.101625	4	0.025406	31.7145	0.0001*
X_1_-pH	0.008427	1	0.008427	10.51935	0.0142*
X_3_-yeast extract	0.01068	1	0.01068	13.33216	0.0082*
X_7_-l-tyrosine	0.072075	1	0.072075	89.97058	<0.0001*
X_8_-CuSO_4_	0.010443	1	0.010443	13.0359	0.0086*
Residual	0.02175	7	0.02175		
Cor total	0.005608	12			

*P* < 0.05, * Significant *P* value

### Box–Behnken Design

Further optimization of the factors that found to be significant from the Plackett–Burman design were carried out which included pH (X_1_), yeast extract (X_3_), l-tyrosine (X_7_), and CuSO_4_ (X_8_). The results obtained were submitted to ANOVA using the Design expert software and results were presented in Table 2 (version 8.0, Stat-Ease Inc. USA), and the regression model equation was given as:

1 $$ \begin{aligned} {\text{L-DOPA}} = & 1. 3 1- 0.0 7 7 {\text{X}}_{ 1} + 0. 1 8 {\text{ X}}_{ 3} + 0. 1 9 {\text{ X}}_{ 7} + 0. 1 5 {\text{X}}_{ 8} - 0. 1 7 {\text{ X}}_{ 1} {\text{X}}_{ 3} - 0. 2 1 {\text{ X}}_{ 1} {\text{X}}_{ 7} - 0. 1 1 {\text{ X}}_{ 1} {\text{X}}_{ 8} \\ & + 0.0 7 1 {\text{ X}}_{ 3} {\text{X}}_{ 7} + 0.0 8 9 {\text{ X}}_{ 3} {\text{X}}_{ 8} + 0. 1 3 {\text{X}}_{ 7} {\text{X}}_{ 8} - 0. 5 4 {\text{ X}}_{ 1}^{ 2} - 0. 3 2 {\text{ X}}_{ 3}^{ 2} - 0. 2 5 {\text{ X}}_{ 7}^{ 2} - 0. 2 8 {\text{ X}}_{ 8}^{ 2} \\ \end{aligned} $$

where X_1_ is pH, X_3_ is yeast extract, X_7_ is l-tyrosine, and X_8_ is CuSO_4_. The ANOVA of the quadratic regression model (Table 2) demonstrated that Eq. (1) is a highly significant model (*P* = 0.001). The model F value of 19.55 implies that the model was significant. The goodness of fit of the model was checked using the determination coefficient (*R*^2^). In this case, the value of the *R*^2^ was 0.9514. The value of the adjusted determination coefficient (Adj *R*^2^ = 0.9027) was in reasonable agreement with the Pred R^2^ (0.7409). The lack-of-fit value (0.1203) for this model was not significant relative to the pure error, which was good to fit the model. “Adeq Precision” measures the signal-to-noise ratio [23]. The “Adeq Precision” ratio of 30.520 obtained in this study indicates an adequate signal. Thus, this model can be used to navigate the design space.

**Table 2:** Analysis of variance (ANOVA) for the fitted quadratic polynomial model of l-DOPA production

Source	Sum of Squares	df	Mean Square	F value	*P* value Prob > F
Model	4.067057	14	0.290504	19.55822	<0.0001*
X_1_-pH	0.070994	1	0.070994	4.779684	0.0463*
X_3_-Yeast extract	0.392047	1	0.392047	26.39459	0.0002*
X_7_-L-tyrosin	0.45202	1	0.45202	30.4323	<0.0001*
X_8_-CuSO_4_	0.262552	1	0.262552	17.67635	0.0009*
X_1_ X_3_	0.110889	1	0.110889	7.465614	0.0162*
X_1_ X_7_	0.178929	1	0.178929	12.04641	0.0037*
X_1_ X_8_	0.05267	1	0.05267	3.54603	0.0806
X_3_ X_7_	0.020306	1	0.020306	1.36712	0.2618
X_3_ X_8_	0.032041	1	0.032041	2.157164	0.1640
X_7_ X_8_	0.069696	1	0.069696	4.692291	0.0480*
X_1_^2^	1.897243	1	1.897243	127.7321	<0.0001*
X_3_^2^	0.667645	1	0.667645	44.9493	<0.0001*
X_7_^2^	0.408899	1	0.408899	27.52919	0.0001*
X_8_^2^	0.514278	1	0.514278	34.6238	<0.0001*
Residual	0.207946	14	0.014853		
Lack of fit	0.186523	10	0.018652	3.482636	0.1203
Pure error	0.021423	4	5.3558		
Cor total	4.275003	28			

*P* < 0.05, * Significant *P* value

### Three-Dimensional (3D) Response Surface Curves

**Fig. 2:**
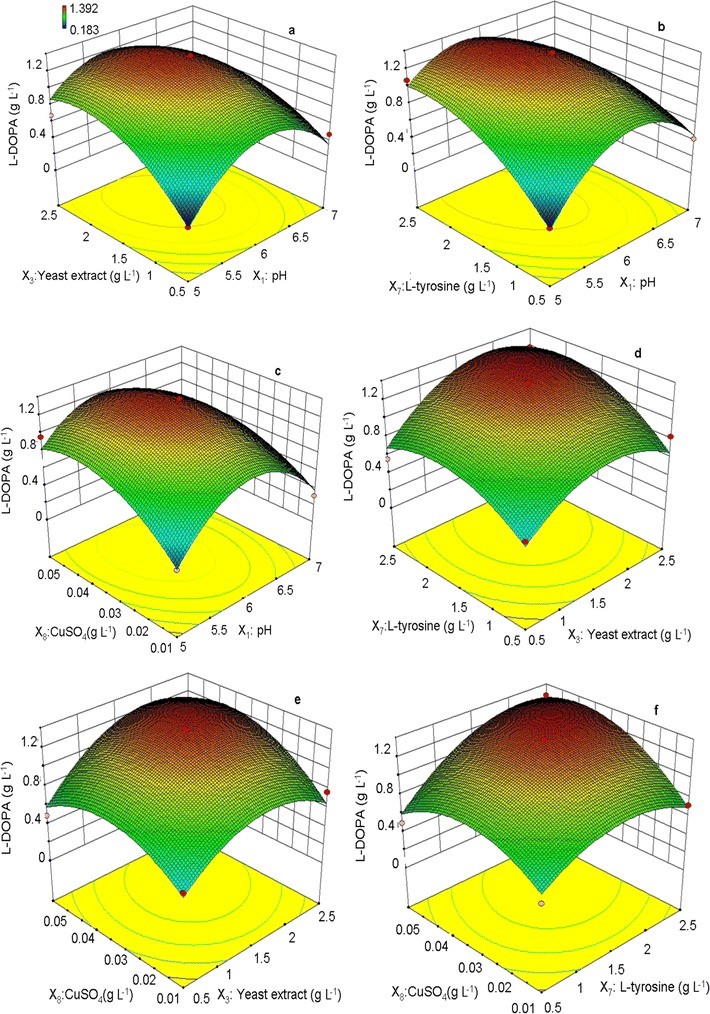
Three-dimensional response surface curve showing the effect of interactions of **a** pH and yeast extract **b** pH and l-tyrosine **c** pH and CuSO_4_**d** yeast extract and l-tyrosine **e** yeast extract and CuSO_4_**f**l-tyrosine and CuSO_4_

3D graphs were generated for the pair wise combination of the four factors while keeping the other two at their optimum levels for l-DOPA production. The graphs are given here to highlight the roles played by various factors in the final yield of l-DOPA. The response surface plot (Fig. 2a) of the interaction of pH and yeast extract indicates that interaction of these components significantly affected the production of l-DOPA. The higher and lower levels of these components affect the l-DOPA yield drastically while mid-levels provide a maximum yield. The interaction between pH and yeast extract was found to significant because acidic and alkaline pH results in lower l-DOPA yields might be because of inhibited tyrosinase activity and cell viability. Also at alkaline pH, less l-DOPA yield resulted due to the conversion of l-DOPA into further metabolites like dopaquinone and melanin [9]. Previous reports shows that Egyptian Black Yeast produced l-DOPA at 10 pH [14], while *Y.* *lipolytica* NRRL-143 and *A.* *oryzae* shows the l-DOPA production at acidic condition; 3.5 and 5.4 respectively [9, 10].

The response surface curve (Fig. 2b) of the interaction between pH and l-tyrosine showed that l-DOPA production was drastically affected by the levels of these factors. The higher and lower concentrations of both factors resulted in lesser l-DOPA yield. The interaction between pH and l-tyrosine was found to highly significant because its solubility is decreases at neutral and alkaline conditions while l-tyrosine soluble at acidic conditions [11, 24]. The higher concentration of l-tyrosine inhibited the l-DOPA production due to its decreased solubility [10, 25].

The interaction between pH and CuSO_4_ less significantly affect the yield of l-DOPA. The statistical analysis showed the insignificant *P* value (0.806) for this interaction (Fig. 2c; Table 2). In addition, the interaction between yeast extract and l-tyrosine (Fig. 2d) found to be insignificant. The effect of the interaction between yeast extract and CuSO_4_ (Fig. 2e) indicates that the l-DOPA yield was not highly altered by changes in the concentration of both media components. The shape of the response surface curve and statistical analysis (Table 2) indicate that highly insignificant interaction occurred between these factors.

The response surface curve of l-tyrosine and CuSO_4_ (Fig. 2f) showed a positive effect on l-DOPA production because the tyrosinase involved in the conversion of l-tyrosine to l-DOPA is a copper-containing enzyme [26]. The use of CuSO_4_ in the media for l-DOPA production by *A.* *rutilum* has been reported earlier [13].

### Validation of the Experimental Model

Validation was carried out under conditions predicted by the model. The optimized levels predicted by the model were pH 6.1, 1.659 g L^−1^ yeast extract, 1.491 g L^−1^l-tyrosine and 0.0290 g L^−1^ CuSO_4_. The predicted yield of l-DOPA with these concentrations was 1.319 g L^−1^, while the actual yield obtained was 1.273 g L^−1^. A close correlation between the experimental and predicted values was observed, which validates this model.

### l-DOPA Yield and Tyrosinase Activity

**Fig. 3:**
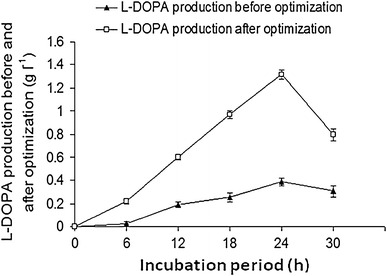
l-DOPA production before and after optimization by RSM

The l-DOPA production before and after optimization is depicted in Fig. 3, which indicates that in the medium before optimization, l-DOPA production started after the 6th hour with a yield of 0.0261 g L^−1^, gradually increased to 0.387 g L^−1^ at the 24th hour, and then decreased to 0.307 g L^−1^ at the 30th hour. In contrast, in the medium optimized by RSM, l-DOPA production started at the 6th hour with a yield of 0.218 g L^−1^, gradually increased to 1.391 g L^−1^ at the 24th hour, and finally decreased to 0.794 g L^−1^ at the 30th hour. The decrease in the l-DOPA yield after the 18th hour was due to the conversion of l-DOPA to further metabolites, such as dopaquinone and melanin [10, 11]. Thus, the medium optimization by RSM resulted in a 3.594-fold increase in the l-DOPA yield over the yield before optimization. The literature survey revealed that single and multiple stage cell suspension cultures of *M.* *pruriens* have been reported to yield 0.028 g L^−1^l-DOPA within 15 and 30 days, respectively [17]. *P.* *grandiflora* has been reported to produce 0.488 g L^−1^ of l-DOPA at the 16th hour [16]; *A.* *rutilum* produced 0.89 g L^−1^l-DOPA, whereas Egyptian black yeast yielded 0.064 g L^−1^ [13, 14]. Thus *Y.* *lipolytica-*NCIM 3450 in the present study produced the highest yield of l-DOPA (1.273 g L^−1^). The *Y.* *lipolytica*-NCIM 3450 reported here produced maximum l-DOPA and has several advantages over the plant, fungal, and bacterial sources used earlier, such as a short incubation period, efficient production, and requirement of simple medium components. The l-DOPA produced previously by bacterial sources like *E.* *herbicola* used pyrocatechol as substrate, which is a toxic phenolic compound, and required polyacrylamide gel, which is an expensive chemical [8, 11]. Thus, the present study contributes to the optimization of the nutritional requirements that will be most useful for large-scale production of l-DOPA using *Y.* *lipolytica*-NCIM 3450. The highest tyrosinase activity was found to be 2738 U mg^−1^. On the other hand, some pycnoporus species *P. sanguineus*, Edible mushroom, bacteria *Thermomicrobium roseum* and yeast *Y.* *lipolytica* NRRL-143 have Specific activity 30, 21.92, 2.49 and 1.55 U mg^−1^ respectively [10, 27–29].

### Analysis of l-DOPA by HPTLC and HPLC

The HPTLC peak profile and the HPTLC plate (Electronic supplementary material Fig. S1) of the cell-free broth showed a distinct peak and band at the RF 0.24, which was identical to standard l-DOPA (0.23). These results primarily confirmed the l-DOPA production in the medium. The HPLC elution profile of standard l-DOPA showed a peak at the retention time 2.723 min (Electronic supplementary material Fig. S2), while the HPLC elution profile of the broth after incubation showed a prominent peak at the retention time 2.721 min. This analysis confirmed the production of l-DOPA.

## Experimental Section

### Chemicals, Strain and l-DOPA Production

l-tyrosine and l-DOPA were purchased from Sigma-Aldrich (St Louis, MO, USA) and all other chemicals were obtained from Himedia (India). The strain *Y.* *lipolytica*-NCIM 3450 was purchased from National Collection of Industrial Microorganism (NCIM), Pune, India. The medium for the cultivation of the *Y.* *lipolytica* strain composed of 1 g L^−1^ yeast extract, 0.5 g L^−1^ peptone, 0.5 g L^−1^ glucose and 1 g L^−1^l-tyrosine at pH 7. The stock cultures of yeast strain were maintained routinely on this medium and stored at 4 °C until used. l-DOPA production was carried out in 250 mL Erlenmeyer flask containing medium mentioned earlier. These flasks were kept in an incubator shaker at 30 °C and 120 rpm for 24 h. l-DOPA was assayed in cell free broth which was obtained after centrifugation at 5000 rpm. The optimization of l-DOPA production was carried out by using Plackett–Burman design and RSM.

### Screening of the Critical Factors Using a Plackett–Burman Design

Plackett–Burman design, an efficient technique for medium component optimization, was used to pick factors that significantly influenced l-DOPA production and insignificant ones were eliminated in order to obtain a smaller, more manageable set of factors. The factors affecting the yield of l-DOPA were selected by screening various carbon sources, nitrogen sources, mineral salts and physical factors such as pH and temperature. In addition, some of these variables were selected from the primary literature review [13, 14]. A total of 11 process parameters, including X_1_ (pH), X_2_ (Temperature), X_3_ (Yeast extract), X_4_ (Peptone), X_5_ (Beef extract), X_6_(Sucrose), X_7_ (l-tyrosine), X_8_ (CuSO_4_), X_9_(MgSO_4_), X_10_ (K_2_HPO_4_), X_11_(Thiamine) were added at two levels: low (−1) and high (+1). The low and high levels of these factors were taken as pH (5 and 7), temperature (20 °C and 50 °C). While levels of media components were (g L^−1^): yeast extract (0.5 and 2.5), peptone (0.5 and 2.5), beef extract (0.5 and 2.5), sucrose (0.5 and 2.5), l-tyrosine (0.5 and 2.5), CuSO_4_ (0.01 and 0.05), MgSO_4_ (0.001 and 0.005), K_2_HPO_4_ (0.5 and 2.5) and thiamine (0.001 and 0.005). The full experimental plan with l-DOPA yield is presented in Electronic supplementary material Table S1. The statistical significance of the first-order model was identified using Fisher’s test for analysis of variance (ANOVA) by Design expert software (version 8.0, Stat-Ease Inc. USA). Moreover, the multiple correlation coefficients (R^2^) were used to express the fit of this first model.

### Optimization by Box–Behnken Design

Based on the results of Plackett–Burman experiments, critical factors were further optimized. The variables each at levels with three replicates at the centre points [23, 30] was used to fit a polynomial model. The experimental plan with l-DOPA yield for Box–Behnken design is given in Electronic supplementary material Table S2. A multiple regression analysis of the data was carried out to define the response in terms of the independent variables. Response surface graphs were obtained to understand the effect of the variables, individually and in combination, and to determine their optimum levels for maximum l-DOPA production by using Design expert software (version 8.0, Stat-Ease Inc. USA). All trials were performed in triplicate, and the average l-DOPA yield was used as response Y.

### l-DOPA Production and Tyrosinase Activity

After validation of the experiment using the optimum process parameters generated by the Design Expert software, the l-DOPA production was observed in the medium before optimization and after optimization. The l-DOPA production was observed at 6-h of time intervals for up to 24 h. The tyrosinase activity was observed at optimum incubation period.

### Analysis of l-DOPA by HPTLC and HPLC

High-performance thin-layer chromatography (HPTLC) analysis of the cell-free broth was performed using a HPTLC system (CAMAG, Switzerland). The conditions used for HPTLC were similar to those in the previously described method [12]. High-performance liquid chromatography (HPLC) analysis of the cell-free broth was carried out (Waters model no. 2690) on a C18 column (4.6 mm × 250 mm, Symmetry) using methanol as mobile phase, with a flow rate of 1 mL min^−1^ for 10 min and a UV detector at 280 nm. The standard l-DOPA and cell-free broth were prepared in HPLC-grade water and injected into the HPLC column [11, 13].

### l-DOPA and Tyrosinase Assay

l-DOPA produced in the broth was determined according to Arnow’s method [25]. The tyrosinase activity was determined by the previously described method [10, 12, 31]. The protein content in the cell free broth was determined using Lowry’s method [32].

## Conclusion

Thus, statistical method not only helped in locating the optimum levels of the most significant factors considered with minimum resources and time but also proved to be useful and satisfactory in this process-optimizing exercise. The optimization of vital nutritional parameters by using RSM significantly enhanced the yield of l-DOPA as proved its feasibility of the process for large scale production by *Y.* *lipolytica*-NCIM 3450. So the *Y.* *lipolytica*-NCIM 3450 can be a potential source for l-DOPA production.

## Electronic supplementary material

Below is the link to the electronic supplementary material.

Supplementary material 1 (DOC 275 kb)
