# Supplementary material for: Evaluation of Various Factors Affecting Bioconversion of l-Tyrosine to l-DOPA by Yeast Yarrowia lipolytica-NCIM 3450 Using Response Surface Methodology
Source: Nat Prod Bioprospect. 2014 May 7;4(3):141–7. doi: 10.1007/s13659-014-0017-3 (PMC4050310; doi:10.1007/s13659-014-0017-3)
Supplement: Supplementary file 1 — Supplementary material 1 (DOC 275 kb) [file 13659_2014_17_MOESM1_ESM.doc]

**Evaluation of various factors affecting Bioconversion of L-tyrosine to L-DOPA by yeast *Yarrowia lipolytica-*NCIM 3450 using Response surface methodology**

Swati T. Gurme^1^, Shripad N. Surwase^2^, Sushama A. Patil^1^, Jyoti P. Jadhav ^1^*

^1^Department of Biotechnology Shivaji University, Kolhapur 416004

^2^Department of Microbiology Shivaji University, Kolhapur 416004

*Corresponding author

Prof. Jyoti P. Jadhav

Head Department of Biotechnology,

Shivaji University, Vidyanagar,

Kolhapur 416004, India

E-mail: jpjbiochem@gmail.com

Tel.: +91 231 2609365

Fax: +91 231 1691533

**Table S1** Design matrix with Observed and predicted response (L-DOPA production) for the experiments performed using Plackett–Burman design

| Standard  order | X_1_ | X_2_ | X_3_ | X_4_ | X_5_ | X_6_ | X_7_ | X_8_ | X_9_ | X_10_ | X_11_ | Actual response (Y (g/l) | Predicated response (Y_1_ g/l ) |
| --- | --- | --- | --- | --- | --- | --- | --- | --- | --- | --- | --- | --- | --- |
| 1 | 1 | 1 | -1 | 1 | 1 | 1 | -1 | -1 | -1 | 1 | -1 | 0.297 | 0.277167 |
| 2 | -1 | 1 | 1 | -1 | 1 | 1 | 1 | -1 | -1 | -1 | 1 | 0.425 | 0.438833 |
| 3 | 1 | -1 | 1 | 1 | -1 | 1 | 1 | 1 | -1 | -1 | -1 | 0.561 | 0.550833 |
| 4 | -1 | 1 | -1 | 1 | 1 | -1 | 1 | 1 | 1 | -1 | -1 | 0.427 | 0.438167 |
| 5 | -1 | -1 | 1 | -1 | 1 | 1 | -1 | 1 | 1 | 1 | -1 | 0.364 | 0.342833 |
| 6 | -1 | -1 | -1 | 1 | -1 | 1 | 1 | -1 | 1 | 1 | 1 | 0.358 | 0.379167 |
| 7 | 1 | -1 | -1 | -1 | 1 | -1 | 1 | 1 | -1 | 1 | 1 | 0.501 | 0.491167 |
| 8 | 1 | 1 | -1 | -1 | -1 | 1 | -1 | 1 | 1 | -1 | 1 | 0.31 | 0.336167 |
| 9 | 1 | 1 | 1 | -1 | -1 | -1 | 1 | -1 | 1 | 1 | -1 | 0.518 | 0.491833 |
| 10 | -1 | 1 | 1 | 1 | -1 | -1 | -1 | 1 | -1 | 1 | 1 | 0.339 | 0.342833 |
| 11 | 1 | -1 | 1 | 1 | 1 | -1 | -1 | -1 | 1 | -1 | 1 | 0.297 | 0.336833 |
| 12 | -1 | -1 | -1 | -1 | -1 | -1 | -1 | -1 | -1 | -1 | -1 | 0.253 | 0.224167 |
| 13 | 0 | 0 | 0 | 0 | 0 | 0 | 0 | 0 | 0 | 0 | 0 | 0.541 | 0.542011 |

-1 low level, +1 high level, 0 center point X_1_ (pH), X_2_ (Temperature), X_3_ (Yeast extract), X_4_ (Peptone), X_5_ (Beef extract), X_6_(Sucrose), X_7_ (L-tyrosine), X_8_ (CuSO_4_), X_9_(MgSO_4_), X_10_ (K_2_HPO_4_), X_11_(Thiamine) Y and Y_1_ (L-DOPA production)

**Table S2** The Box-Behnken design matrix for coded variables along with actual and predicted responses for L-DOPA production

| Standard order | Factor  X_1_ | Factor  X_3_ | Factor  X_7_ | Factor  X_8_ | Actual response (Y g/l ) | Predicated response (Y_1_ g/l ) | Externally studentized Residual |
| --- | --- | --- | --- | --- | --- | --- | --- |
| 1 | -1 | -1 | 0 | 0 | 0.183 | 0.182417 | 0.007145 |
| 2 | 1 | -1 | 0 | 0 | 0.479 | 0.361583 | 1.568426 |
| 3 | -1 | 1 | 0 | 0 | 0.681 | 0.876917 | -3.21548 |
| 4 | 1 | 1 | 0 | 0 | 0.311 | 0.390083 | -1.00567 |
| 5 | 0 | 0 | -1 | -1 | 0.475 | 0.57175 | -1.25481 |
| 6 | 0 | 0 | 1 | -1 | 0.719 | 0.695917 | 0.283622 |
| 7 | 0 | 0 | -1 | 1 | 0.502 | 0.603583 | -1.32575 |
| 8 | 0 | 0 | 1 | 1 | 1.274 | 1.25575 | 0.223976 |
| 9 | -1 | 0 | 0 | -1 | 0.293 | 0.30625 | -0.16246 |
| 10 | 1 | 0 | 0 | -1 | 0.308 | 0.381917 | -0.93538 |
| 11 | -1 | 0 | 0 | 1 | 0.961 | 0.831583 | 1.764969 |
| 12 | 1 | 0 | 0 | 1 | 0.517 | 0.44825 | 0.866075 |
| 13 | 0 | -1 | -1 | 0 | 0.492 | 0.438917 | 0.661058 |
| 14 | 0 | 1 | -1 | 0 | 0.835 | 0.657917 | 2.715456 |
| 15 | 0 | -1 | 1 | 0 | 0.563 | 0.684583 | -1.6353 |
| 16 | 0 | 1 | 1 | 0 | 1.191 | 1.188583 | 0.029603 |
| 17 | -1 | 0 | -1 | 0 | 0.206 | 0.193833 | 0.149157 |
| 18 | 1 | 0 | -1 | 0 | 0.419 | 0.463 | -0.54508 |
| 19 | -1 | 0 | 1 | 0 | 1.072 | 1.005 | 0.842808 |
| 20 | 1 | 0 | 1 | 0 | 0.439 | 0.428167 | 0.132788 |
| 21 | 0 | -1 | 0 | -1 | 0.517 | 0.472833 | 0.547194 |
| 22 | 0 | 1 | 0 | -1 | 0.772 | 0.655333 | 1.556534 |
| 23 | 0 | -1 | 0 | 1 | 0.496 | 0.589667 | -1.21023 |
| 24 | 0 | 1 | 0 | 1 | 1.109 | 1.130167 | -0.25994 |
| 25 | 0 | 0 | 0 | 0 | 1.285 | 1.3144 | -0.26057 |
| 26 | 0 | 0 | 0 | 0 | 1.391 | 1.3144 | 0.68941 |
| 27 | 0 | 0 | 0 | 0 | 1.231 | 1.3144 | -0.75317 |
| 28 | 0 | 0 | 0 | 0 | 1.392 | 1.3144 | 0.698745 |
| 29 | 0 | 0 | 0 | 0 | 1.273 | 1.3144 | -0.36788 |

-1 low level, +1 high level, 0 center point; X_1_ (pH), X_3_ (Yeast extract), X_7_ (L-tyrosine), X_8_ (CuSO_4_); Y (L-DOPA production)


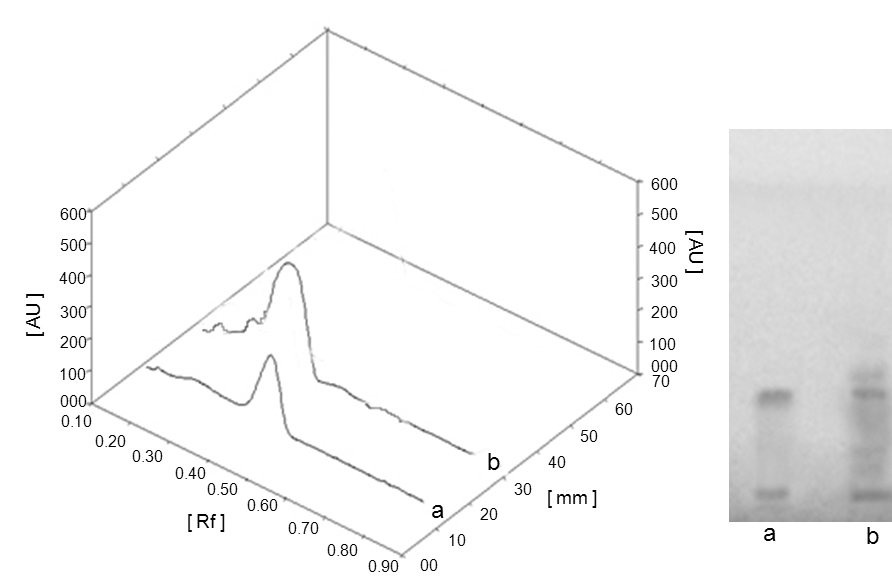


**Fig.** **S1** HPTLC analysis (a) standard L-DOPA (b) broth after incubation


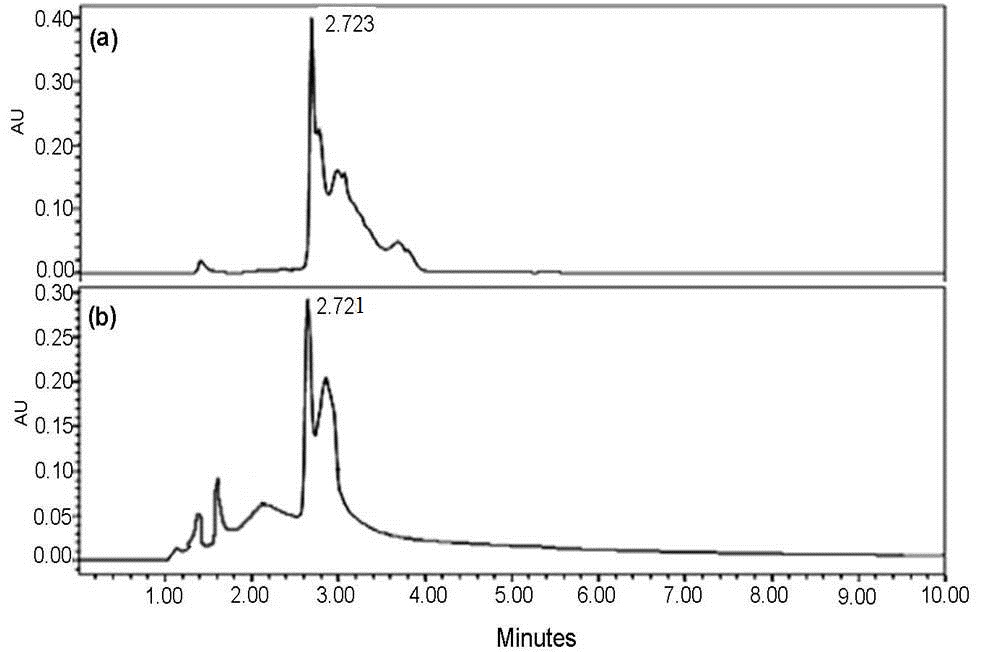


**Fig. S2** HPLC elution profile (a) standard L-DOPA and (b) produced L-DOPA
